# Dormancy in Colorectal Carcinoma: Detection and Therapeutic Potential

**DOI:** 10.3390/biom15081119

**Published:** 2025-08-04

**Authors:** Sofía Fernández-Hernández, Miguel Ángel Hidalgo-León, Carlos Lacalle-González, Rocío Olivera-Salazar, Michael Ochieng’ Otieno, Jesús García-Foncillas, Javier Martinez-Useros

**Affiliations:** 1 Department of Pathology, Skien Hospital, Vestfold og Telemark, Ulefossvegen 55, 3710 Skien, Norway; sofher@sthf.no; 2 Translational Oncology Division, OncoHealth Institute, Health Research Institute Fundación Jimenez Diaz, Fundación Jimenez Díaz University Hospital, Universidad Autónoma de Madrid (IIS-FJD, UAM), 28040 Madrid, Spain; m.angelhl@hotmail.com (M.Á.H.-L.); c.lacalle92@gmail.com (C.L.-G.); mikeotson@yahoo.com (M.O.O.); jgfoncillas@quironsalud.es (J.G.-F.); 3 New Therapies Laboratory, Health Research Institute-Fundación Jiménez Díaz University Hospital (IIS-FJD), Avda. Reyes Católicos, 2, 28040 Madrid, Spain; rocio.olivera@quironsalud.es; 4 Department of Medical Oncology, Fundación Jiménez Díaz University Hospital, 28040 Madrid, Spain; 5 Area of Physiology, Department of Basic Health Sciences, Faculty of Health Sciences, Rey Juan Carlos University, 28922 Madrid, Spain

**Keywords:** colorectal cancer, dormant cells, metastasis, liquid biopsy, quiescence

## Abstract

Colorectal cancer (CRC) is not only the third most common cancer worldwide, with 1.1 million new cases per year; it is also the second leading cause of cancer death. However, mortality has decreased since 2012 due to early detection programs and better therapeutic approaches. While many patients are diagnosed at an early stage, there is up to 50% relapse after optimal initial treatment. Therefore, it is crucial to explore the mechanism underlying the development of recurrences and metastasis. It is known that tumors release dormant cells that escape chemotherapy and nest in a target organ without proliferating. Under certain circumstances that are not yet entirely clear, they can be activated and metastasize. Therefore, the objective of this work is to explore the detailed mechanisms of dormancy, including early detection of recurrence and therapeutic approaches for the treatment of CRC. The specific objectives are to determine biomarkers that may be useful in identifying dormant cells to detect minimal residual disease (MRD) after surgery and predicting disease progression, as well as evaluating biomarkers that are susceptible to therapeutic intervention.

## 1. Introduction

CRC is the third most commonly diagnosed cancer and the second leading cause of cancer death worldwide. It occurs most often in developed countries with upper-middle incomes. This may be associated with known risk factors, such as alcohol intake, tobacco use, obesity, sedentary lifestyle, and dietary patterns (diets low in fruits, vegetables, and unrefined plant foods, and diets high in red meat, processed foods, and fats). In Spain, it represents the second incidence in both sexes and the second cause of cancer mortality [1,2]. Although the average 5-year survival rate for patients with CRC has risen to about 64%, the survival rate for patients with advanced disease is less than 15%. Disseminated disease is present in approximately 50% of cases at the time of diagnosis. This leads to lower survival since patients in these circumstances would no longer be candidates for surgical treatment [3]. Staging and decision-making are primarily based on surgical pathology. However, this provides imprecise estimates of MRD following surgery with curative intent. In addition, staging itself does not predict the potential effect of adjuvant therapy. Using staging as the sole criterion may lead to the undertreatment of individuals with MRD or the overtreatment of patients already cured by surgery. Therefore, a better definition of MRD in CRC is required after surgical resection with curative intent, as the early detection of relapses could improve survival in these cases [2].

Neoadjuvant chemotherapy is proposed as the initial treatment in most patients with metastatic colorectal cancer (mCRC) with initially unresectable liver metastases. In addition, the sensitivity of the tumor to chemotherapy and tumor biology can be evaluated by the degree of de-escalation that can be achieved after treatment. This therapeutic regimen is determined by the percentage of patients who achieve an R0 complete resection margin (resected tumor with tumor-free margins) [4]. Surgical resection R0 of the CRC with liver metastases is a potentially curative treatment, with 5-year survival rates of 20–45%. The R0 resectability criteria of the mCRCs are mainly based on technical criteria. However, there is no evidence of the benefit of adjuvant chemotherapy in patients with mCRC after R0 surgery of metastases. Additionally, chemotherapy is not administered until the disease progresses. That is why early detection of recurrence prior to imaging detection, according to RECIST (Response Evaluation Criteria In Solid Tumors) criteria through one-dimensional measurements obtained with different diagnostic methods, such as CT, MRI, or PET, is of crucial importance in the control and management of patients with mCRC [4].

The choice of neoadjuvant chemotherapy treatment is determined by biological determinants, such as the mutational status of RAS (*KRAS* and *NRAS*) and *BRAF* and the status of base-pairing error repair proteins dMMR/MSI. Since these mutations are negative predictors for the use of monoclonal antibodies (mAb) against the epidermal growth factor receptor (EGFR), the study of mutations in *KRAS* and *NRAS* is mandatory before starting treatment. It is also recommended that MMR and the mutational study of *BRAF* be studied in all patients at the time of diagnosis of mCRC. This can be carried out in both the primary tumor and metastasis, with a suggested response time of 10 days [1]. The currently recommended follow-up programs, which consist of imaging techniques and the control of carcinoembryonic antigen (CEA) in plasma, are suboptimal: they do not detect MRD, and mostly, they diagnose only relapses in very advanced stages. It is, therefore, necessary to identify biomarkers for early detection of recurrence more efficiently [2].

Thus, recurrence and metastasis in cancer, after apparently successful treatment, constitute a threat in clinical oncology and are the leading causes of death among patients. This phenomenon is mainly due to the fact that disseminated tumor cells remain dormant, a viable but non-proliferating state, for an extended period. These cells enter the cell cycle in the G0/G1 phase, in which they consume less glucose and are in a state of metabolic suppression to adapt to their new microenvironment through epigenetic modifications. It is, therefore, a reversible situation once the stimuli inducing this state are eliminated [4]. All this makes them resistant to treatments and “invisible” to immune surveillance. Subsequently, under favorable conditions, the disseminated dormant tumor cells “wake up” and resume their proliferation [4]. Hence, the detection of these quiescent or slow-cycling cells is a challenge.

Several publications focus on the search for possible indicators of cell dormancy by studying genes related to cell cycle arrest, proliferation, stemness, epithelial-mesenchymal transition (TEM), and hypoxia regulation [5]. Liquid biopsy as a method of detecting these cells has recently gained important relevance as an alternative and non-invasive complementary technique to tissue biopsy in cancer patients. As technological advances have improved both feasibility and response time, liquid biopsy as a minimally invasive procedure has allowed longitudinal disease evaluation. Tumor molecular analysis has been expanded with the recognition of spatial and temporal heterogeneity, and many limitations of traditional tissue biopsy have been overcome [6]. At the moment, what has proven to be an accurate prognostic biomarker in several types of tumors, CRC among them, is the detection of circulating tumor cells (CTC) and the analysis of circulating tumor DNA in plasma (ctDNA), which seems to represent a better estimate of MRD in patients after surgical treatment [2].

This study focuses on newly diagnosed patients with potentially resectable metastatic colorectal cancer, where neoadjuvant chemotherapy is followed by curative-intent surgery and negative margins of resection (R0 resection).

The primary aim of this review is to propose a molecular signature that would enable us to identify circulating dormant tumor cells. This strategy would facilitate the detection of these cells in patients at risk of early relapse or recurrent malignant disease (RMD) and those likely to be detected by liquid biopsy in peripheral blood. To this end, our specific objectives are to provide the molecular mechanism of dormancy by tumor cells, the specific factors that they present, and methods that can be used to detect these cells by liquid biopsy. We will then propose therapies, which could be effective to eradicate these cells.

## 2. Theoretical Foundation

### 2.1. Current Status: Cellular Dormancy

**Figure 1:**
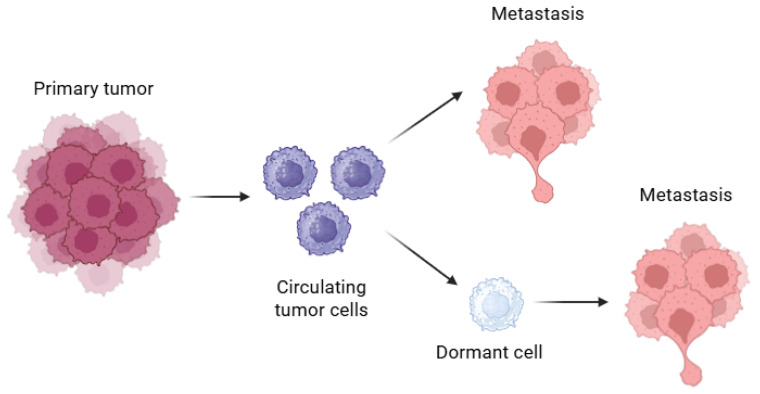
Dormancy and metastasis. Circulating tumor cells (CTCs) (purple), with some dormant (CD), representing the origin of metastasis.

There are many theories currently available to explain the mechanism of cancer recurrence. The tumor dormancy theory holds that micrometastatic lesions already exist when the primary cancer is diagnosed. These dormant cancer cells can remain relatively stable for months or years. The micrometastatic foci are stable and are too small to be detected by imaging tests. Hence, the patient appears to be disease-free [7] (Figure 1).

Tumors grow when unknown factors alter the microenvironment or immune surveillance fails to inhibit dormant cancer cells. Cancer recurrence can be confirmed when tumors grow large enough to be detected or cause symptoms [7]. While the concept of dormant tumor cells has already been defined, it is important to redefine the term dormancy since it often refers to different phenomena.

A form of dormancy refers to the tumor mass and corresponds to the cessation of growth (or constant size) of a tumor, whether a primary or metastatic tumor. Although some tumor cells, in this case, are proliferating, the tumor mass does not expand due to an equivalent rate of cell death. This cell death is usually due to hypoxia and limited vascularization, which is often referred to as “angiogenic dormancy”. The cell death can also be due to a continuous elimination of proliferating cells by the immune system, which is called “immune dormancy” [8]. Quiescence is a specific, reversible state of cellular dormancy characterized by the arrest of the cell cycle in the G0 phase, where cells remain metabolically active but do not proliferate. In contrast, dormancy is a broader concept encompassing various non-proliferative states of tumor cells or tumor masses, including quiescence, but also conditions where tumor growth is limited by microenvironmental factors, such as angiogenic suppression or immune surveillance mentioned above. Thus, while all quiescent cells are dormant, not all dormant states are due to quiescence. Understanding this distinction is critical for developing therapeutic strategies aimed at targeting dormant tumor cells to prevent relapse and metastasis [9].

Our interest is limited to the concept of “cellular dormancy”. As already indicated, it corresponds to a reversible state of non-division achieved by tumor cells that try to adapt and survive in changing environments capable of escaping chemotherapy [10,11]. It was postulated that dormant or quiescent tumor cells could be due to pre-existing subclones carrying mutations that allowed them to resist treatments. However, it seems that dormant tumor cells do not correspond to pre-existing tumor populations but to cells with non-mutational mechanisms that allow the cell to acquire the ability to enter this state of dormancy as an adaptive strategy to the stress caused by treatments [12]. Cancer cells enter a reversible state of drug tolerance. Thus, they evade death from chemotherapy and targeted treatments. This reversible state of drug tolerance corresponds to a mechanism conserved during development, which tumor cells have acquired for survival [13].

Numerous studies have examined the possible mechanisms that cause these cells to remain dormant. All of them are complex processes mediated by, among other factors, the interaction of the tumor with the tumor microenvironment [14]. The remodeling and degradation of the extracellular matrix (ECM) are necessary in the early stages of the metastatic cascade. In addition, molecules in the ECM are important not only for the development of metastases, but also for their growth and the possible development of tumor cell quiescence [15]. This process is closely linked to the effects of surgery, which can activate dormant micrometastases and accelerate metastatic progression by allowing tumor cells to enter the bloodstream or lymphatic system, increasing CTCs. Furthermore, surgical stress triggers the release of neuroendocrine mediators that elevate immunosuppressive and proinflammatory cytokines, promoting tumor invasion, metastasis, and the formation of pre-metastatic niches (PMNs). Together, these factors create a microenvironment conducive to metastatic outgrowth following surgery [16].

The immune system plays a dual role in cell dormancy and reactivation. Chronic inflammation, driven by factors like infection, smoking, and neutrophils, can reactivate dormant disseminated cancer cells and promote metastasis. Conversely, immune responses, particularly from CD8+ and CD4+ T cells, can maintain dormancy by preventing metastatic growth or inducing dormancy through cytokine signaling. However, cancer cells may evade immune control by using mechanisms such as MHC-I downregulation, allowing tumor progression under certain conditions [17].

In addition, immunosuppression can trigger the activation of dormant tumor cells. For instance, in rats injected with colorectal cancer cells, metastases did not form unless immunosuppression was induced with cyclosporine A, which led to widespread metastasis [18]. Consequently, radiation therapy may awaken dormant cells by damaging tumor vessels, inducing EMT, and causing immunosuppression, thereby promoting tumor growth and spread [16].

Hypoxic stress plays a fundamental role in cancer progression. However, the mechanism by which hypoxia causes tumors to become more aggressive or metastatic and adapt to adverse environmental stress is not yet well understood. What has been observed is that the gene CSN8 could be a key toggle switch that controls hypoxia-induced malignant tumor progression [19]. In addition to hypoxia and ECM factors, genes related mainly to cell cycle, proliferation, and stem-like features are among the different mechanisms associated with cellular dormancy. The following genes have been studied: FBX8 [5], the imbalance between P38/ERK [20,21], and the transcription factor NANOG [11,22].

Another pathway involved is the Rb-E2F pathway, which is essential at the restriction point where cells proliferate, enter dormancy, or undergo senescence. E2F maintains proliferation independently of external signals and acts as a binary switch between dormancy and proliferation. The depth of dormancy can be regulated by differential gene expression within this pathway, suggesting that dormant cancer cells (DCCs) may have distinct dormancy states compared to healthy dormant cells [17].

### 2.2. Current Status: Liquid Biopsy

Currently, decision-making on the most appropriate treatment for patients with mCRC is based on the following methods: histological study and genotyping of the tumor performed in the biopsy or the tumor resection specimen. Tumor biopsy requires an invasive procedure and only provides a single, static view of a small tumor fragment [18,23]. CEA is one of the blood biomarkers that are applied in clinical practice to evaluate the response to treatment, surveillance, and diagnosis in CRC. However, CEA is an indirect measure of a tumor’s activity. Therefore, a more direct analysis of the disease is needed [24].

Thus, liquid biopsy is emerging as a promising, non-invasive alternative, with the potential to improve current clinical practice in oncology by providing real-time information on a patient’s disease status and response to treatment [24]. In addition, a complete molecular profile of the tumor can be obtained. In this way, the study of mutations of *KRAS*/*NRAS* and *BRAF* mutations, amplifications of *HER2*, microsatellite instability (MSI), and the protein status of base-matching errors (MMRs) repair using minimally invasive and easily accessible techniques could greatly improve decision-making for better patient management [23,24].

Hence, liquid biopsy is being widely studied in mCRC, thanks to its diagnostic, prognostic, and predictive value. As personalized medicine is increasingly important in cancer treatment, new genetic biomarkers are needed to diagnose the emergence of early resistance to current therapies. CTC and ctDNA determination are the most widely adopted approaches due to their high clinical relevance in CRC [6]. ctDNA refers to the proportion of total circulating free DNA (cfDNA) secreted into the bloodstream by tumor cells. According to some studies, it typically constitutes a small fraction: less than 1%. However, with advancements in assay techniques that offer greater sensitivity, ctDNA analysis is rapidly gaining acceptance as a reliable tool in oncology [25]. As aforementioned, tissue biopsy, on the contrary, is not only an invasive procedure, but it is also, in most cases, unable to fully capture tumor heterogeneity and evolution. CTC and ctDNA analysis offer a non-invasive alternative to repeatedly assess the genomic profile of the tumor [25]. Therefore, in addition to genotyping tumors to direct treatment, ctDNA analysis allows them to track their evolution in real-time and detect MRD early in the follow-up of mCRC. The elevation of its levels and the alterations of its mutation and methylation profiles in cancer also make it a promising biomarker [23].

Monitoring the response to treatment during chemotherapy is very important to predict the course of the disease. Thus, in addition to ctDNA and CTC, other biomarkers analyzed in liquid biopsy, such as non-coding RNA and extracellular vesicles (EVs), are being studied. MicroRNAs (miRNAs) appear to play an important role in cancer. They can be encapsulated in EVs, released into biological fluids, and communicate between cells, transferring genetic information. Most of these biomarkers have shown promising results in their clinical application, especially with regard to the study of tumor heterogeneity in relation to response to treatment, the prediction of the appearance of metastasis, and the detection of MRD [26,27]. It is important to note that MRD is a clinical term referring to the overall burden of residual cancer cells remaining after treatment, whereas quiescent cells describe a specific biological state characterized by non-dividing but viable cells.

The use of biologic drugs, such as anti-EGFR and anti-VEGF, in addition to standard chemotherapy, has increased progression-free survival and overall survival for patients with mCRC. However, these benefits have only been observed in a few patients. Currently, no precise biomarkers can explain this disease’s heterogeneity and help with treatment selection. Real-time drug resistance monitoring may offer the opportunity to guide new therapies, such as anti-RAS agents, treatments directed against *BRAF* V600 or *HER2*, and ICIs (immune checkpoint inhibitors). Monitoring with NGS (Next-Generation Sequencing) in the plasma of CRC patients allows for the detection of low-frequency mutations with high sensitivity and the identification of clones with potentially treatable alterations. These aspects are crucial to continuously monitoring patients with mCRC [28].

However, according to a meta-analysis on the value of CTC detection in the follow-up of postoperative recurrence and CRC metastasis, a unified view of this is not yet observed, especially in stages I–III. There are still controversies regarding the follow-up of the postoperative recurrence and metastasis of CRC. Although most of the literature has affirmed the importance of CTCs in the follow-up of postoperative recurrence, metastasis, and prognosis of CRC, some publications still oppose this view [29].

## 3. Characterization of Dormant Cells

Rapid advances have emerged in recent years, especially in targeted therapy and immunotherapy. However, metastasis and relapse, which can occur years or even decades after tumor eradication, remain a major problem in cancer treatment. As already mentioned, it is partly attributed to the induction of the quiescence state of tumor cells. Cancer treatment can eradicate most cancer cells at the primary site, but some cells can go into a resting state and stop proliferating to survive, which is considered one of the causes of MRD [30].

Dormant tumor cells show some characteristics similar to cancer stem cells. This could indicate that the regulation of the state of tumor cell dormancy depends more on mechanisms of epigenetic regulation or adaptive response to stress than on mutational mechanisms. The life cycle of quiescent tumor cells is related to both intracellular signaling and extracellular stimuli. Therefore, the control of the cycle of these cells also seems to respond to a stress-dependent mechanism [30].

In normal cells, reactive oxygen species (ROS) and antioxidants are in balance (redox homeostasis), while increased ROS levels are considered a hallmark of cancer cells. The increase in ROS levels leads to genomic instability, facilitating cell proliferation, increasing motility, and activating oncogenic signaling. Excess ROS produced by chemotherapy can induce irreversible cell damage and lead to tumor cell death. However, some tumor cells may remain dormant by overexpressing antioxidant enzymes to maintain low ROS levels, even lower than normal cells, to evade chemotherapy-induced cell death [30]. Thus, there are different mechanisms by which tumor cells enter a state of dormancy (Table 1).

With regard to epigenetic regulation, one of the studies identified the existence of a primitive non-genetic program that governs dormancy, which could be common in different types of tumors. In this sense, the TET2 protein was studied as a key factor that controls the dormancy or slow cell cycle state, inducing survival and tumor recurrence. TET2 modulates the expression of TNF-α signaling components and restricts pro-apoptotic signaling. It is an epigenetic dioxygenase responsible for the enzymatic oxidation of genomic 5-methylcytosine (5 mC) to 5-hydroxymethylcytosine (5 hmC). In this study, it was shown that 5 hmC could be used not only as a possible epigenetic biomarker that could predict relapse and worse survival of patients, but also as a potential pharmacological target for the elimination of these cells with increased survival [31]. These findings were corroborated by an immune-histochemical study in a cohort of 83 cases of CRC, where cells accumulating high levels of 5 hmC also had overexpression of the TET2 protein. 5 hmC-positive cells were also confirmed to be negative for the Ki67 proliferation marker in these tumors. In addition, a higher proportion of 5 hmC-positive cases was detected in liver metastases (38%) compared to primary tumors (18%) in a cohort of 197 patients with CRC [27,31]. Therefore, 5 hmC could be a possible biomarker in these dormant cells and an indicator of early recurrence.

**Figure 2:**
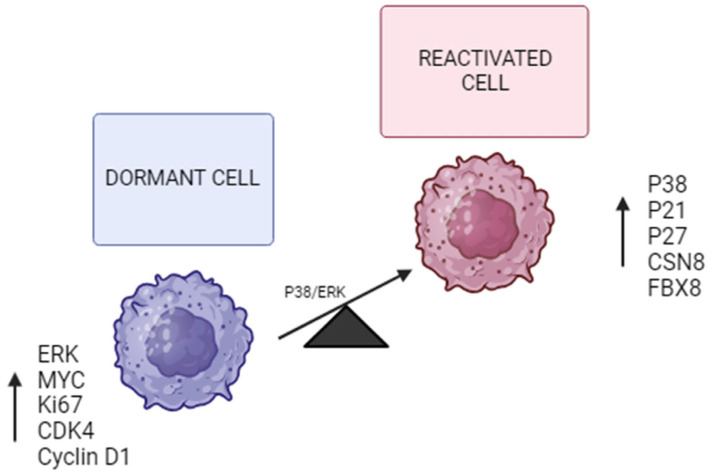
Dormancy vs. cell reactivation genes. Balance sheet for P38/ERK. The imbalance between P38/ERK in favor of P38 induces and maintains a dormancy state through the expression of P21 and P27. The overexpression of factors such as CSN8 and FBX8 is also involved in the induction and maintenance of such a state. On the contrary, the expression of ERK and genes involved in proliferation and cell cycle activation is increased in the activated cell.

Another important mechanism by which tumor cells enter dormancy is a decreased P38/ERK ratio. According to recent studies, a possible signaling crossover between the mitogen-activated protein kinase P38 and ERK1/2 was observed in the induction of the dormant state of tumor cells [20,21]. There are three subfamilies of mitogen-activated protein kinases (MAPKs) in mammals: the ERK, JNK, and P38 kinases. The P38 family consists of four isoforms (α, β, γ, and δ). They have been shown to play important roles in cell proliferation by activating the G1/S and G2/M checkpoints. Additionally, p38α may promote growth arrest by suppressing Cyclin D1 expression and activating the P53 to P21 and/or P16 to RB pathways, among others [20,30]. The involvement of the p38 protein in metastasis and chemoresistance has been shown in recent studies. P38 signaling allows these dormant tumor cells to resist chemotherapy by activating survival mechanisms driven by the overexpression of the protein kinases PERK and BiP that prevent the activation of BAX, a mitochondrial pro-apoptotic protein. P38 activation can also act through other pathways. For example, high levels of P38 result in the expression of CDC42, which induces P21/P27. As already mentioned, P21/P27 repress the expression of Cyclin D1, causing the arrest of the cell cycle [32] (Figure 2).

Another study showed that CSN8 expression was significantly increased in CRC tissues, which is correlated with lymph node metastases and predicted a worse survival rate for patients. The overexpression of CSN8 induces the epithelial-mesenchymal transition (EMT) process in CRC cells, increasing migration and invasion. In addition, it also stopped cell proliferation, increased key dormancy markers, such as NR2F1, DEC2, and P27, and hypoxia response genes, such as HIF-1α and GLUT1. Cell survival was drastically improved under treatment conditions with hypoxia, serum deprivation, or 5-fluorouracil. CSN8 regulated TEM and dormancy, in part, by activating the HIF-1α signaling pathway, which increased HIF-1α mRNA expression by activating NF-κB and stabilizing the HIF-1α protein through its deubiquitination [19].

CSN8 silencing also blocked TEM and hypoxia-induced cellular dormancy processes in vitro and undermined the adaptive capacity of CRC cells in vivo. Thus, CSN8 endows primary CRC cells with highly aggressive and metastatic capabilities and adaptive mechanisms by regulating TEM and hypoxia-induced dormancy. CSN8 could serve as another potential prognostic biomarker in CRC and an ideal target for the elimination of disseminated quiescent cells, thereby reducing the risk of tumor metastasis and recurrence and chemoresistance prevention [19].

Another gene significantly correlated with hypoxia and associated with dormancy, the cell cycle, and MYC pathways is FBX8 (F-Box protein 8). FBX8 is a member of the ubiquitin protease family. In this study, we examined whether the dysregulation of FBX8 was involved in this state by modulating HIF-1α, CDK4, and c-MYC proteins. To this end, the expression of markers related to the induction of cell dormancy was examined, such as CK, E-cadherin, SOX-2, and CD133, along with markers related to cell activation, including Vimentin, Cyclin-D1, Ki-67, C-MYC, and VEGF, in subcutaneous tumors formed by cell lines established in nude mice. The expression of markers related to the induction of cell dormancy was significantly higher in FBX8-overexpressed tumors than in control tumors. In contrast, the expression of markers related to activation or exit from the dormant state increased in tumors lacking FBX8 expression [5].

**Figure 3:**
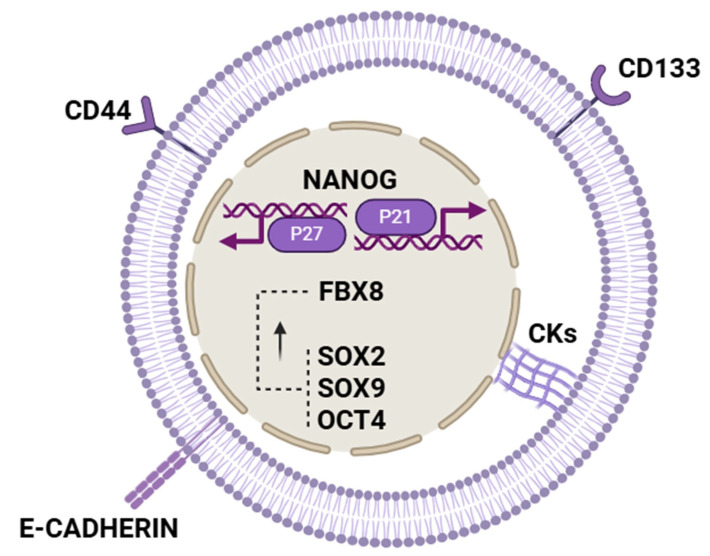
Core genes involved in dormancy. Genes expressed in stem cells that are also expressed in dormant cells (CD44, CD133, SOX2, SOX9, OCT4, and FBX8). The transcription factor NANOG has been overexpressed in dormant cells, inducing dormancy by increasing the transcription of P21 and P27. FBX8 is associated with the upregulation of dormancy-related markers such as SOX2, SOX9, E-cadherin, cytokeratins (CKs), CD44, and CD133. However, the precise molecular mechanisms by which FBX8 regulates the expression of SOX2, SOX9, and OCT4 remain unclear and require further investigation.

Thus, based on the findings of this study, FBX8 appears to modulate cellular dormancy in vitro primarily by regulating HIF-1α, CDK4, and c-MYC. Furthermore, FBX8 is linked to the upregulation of stem cell-related markers such as SOX2, CD44, and CD133, although the precise mechanisms driving this upregulation remain to be elucidated (Figure 3). Thus, a study demonstrated that FBX8 promotes metastatic dormancy in colorectal cancer by ubiquitinating HIF-1α, CDK4, and c-Myc, thereby maintaining G_0_/G_1_ arrest in liver metastases in mouse models. It was also observed in vivo that maintaining FBX8 overexpression prolonged the state of cell dormancy due to an increase in the expression of SOX9 and OCT4 proteins, in addition to those mentioned above (SOX2, CD133, and CD44) and a decrease in the expression of Vimentin, Cyclin-D1, VEGF, HIF-1α, CDK4, and c-MYC. In addition to reducing the expression of these proteins, FBX8 may also promote their degradation through ubiquitination [5]. Given all this, one could also consider FBX8 as a factor that might open a window for possible therapeutic approaches.

Since it seems there could be regulatory pathways common to different tumors that would lead to this state of cellular inactivation, a shared “dormant signature” was identified between tumor cells of non-small cell lung cancer (NSCLC) and CRC to characterize dormant cells. Taken together, it seems that these results could be the subject of future therapeutic interventions. Recent studies compared dormant tumor cells of NSCLC and CRC xenografts ex vivo and gene expression profiles. Principal component analysis revealed a specific component that discriminated inactive and replicative phenotypes in NSCLC and CRC. The discriminatory component showed significant overlap, with 688 genes in common, including ZEB2, a major regulator of stem cell plasticity and TEM. NSCLC and CRC dormant tumor cells were observed to have increased expression of factors related to stemness, TEM, TGF-β, cell adhesion, and chemotaxis, and proliferating cells overexpressed MYC targets and factors involved in RNA metabolism [10].

Thus, as has already been observed, quiescent cells in CRC are characterized by a higher expression of factors related to stemness-like features, such as the transcription factor NANOG (Figure 3). One of the most recent studies focuses on the study of this factor and its relationship with the mechanisms that regulate the entry of dormant tumor cells. The transcription factor NANOG is important for maintaining the state of self-renewal and pluripotency of stem cells. Since dormant tumor cells share mechanisms similar to those of quiescent cancer stem cells, the correlation between dormancy state and NANOG factor in CRC was studied. In this study, serum deprivation was used to induce cell dormancy. It was observed that the regulation of NANOG depended on the fatty acid oxidation pathway (FAO)/ATP citrate lyase (ACLY). NANOG was identified to be overexpressed in CRC dormant cells, and its downregulation could reverse the dormancy state of serum-deprived CRC cells. NANOG overexpression could induce dormancy in CRC Cells by increasing P21 and P27 transcription [11].

Research in colorectal cancer cells shows that NANOG, via fatty acid oxidation/ACLY signaling, induces dormancy under serum deprivation conditions [11]. In addition, NANOG overexpression has also been linked to an increased risk of liver metastases and a worse prognosis for CRC patients. Therefore, it could be an important factor for prognosis and the detection of dormant cells [11,22].

Four other genes related to cancer stem cells (CSCs) have also been identified in the CRC in dormant cells: pluripotency factor KLF4, AXIN2, LGR5, and BMI1. BMI1 was previously identified as the marker of a quiescent intestinal stem cell (ISC) subpopulation, while LGR5 can be found in both proliferating and slow-cycle or chemoresistant ISCs. These findings indicated that in CRC, quiescent cancer cells are enriched in cancer stem cells [10]. Another important aspect that dormant tumor cells also seem to share with cancer stem cells is redox-state-dependent intrinsic signaling pathways, such as the Wnt, Notch, and Hedgehog pathways. Thus, it has been seen that itraconazole interrupts the state of cellular dormancy in the CRC by inhibiting the Wnt pathway [30].

From the aforementioned, in addition to the factors that have been seen to be involved in the process of inducing cell dormancy, quiescent tumor cells and cancer stem cells seem to share the same signaling mechanisms in part. If we consider this premise, it could be argued that the same markers used in detecting stem cells would also allow for the detection of dormant cells through liquid biopsy. Thus, liquid biopsy will help to detect MRD and relapses early.

**Table 1:** Main molecular mechanisms of dormancy.

Mechanism	Description	Key Molecules/Markers	Role in Dormancy	References
Epigenetic regulation.	Non-genetic program controlling dormancy via DNA modification and gene expression regulation.	TET2 protein, 5-hydroxymethylcytosine (5 hmC).	Maintains slow cell cycle state; modulates TNF-α signaling; restricts pro-apoptotic signals; biomarker of dormancy and relapse risk.	[27,31]
MAPK signaling pathway.	Balance between P38 and ERK signaling influences dormancy induction.	P38, ERK 1/2, Cyclin D1, P53, P21, P16.	High P38/ERK ratio promotes growth arrest and survival; represses Cyclin D1; activates cell cycle checkpoints.	[20,21,32,33]
Stress response and chemoresistance.	Activation of survival pathways under stress conditions, including chemotherapy, hypoxia, serum deprivation.	PERK, BiP, BAX, CSN8, HIF-1α, NF-κB.	CSN8 promotes migration, invasion, dormancy markers (NR2F1, DEC2, P27) and hypoxia response; protects cells from apoptosis.	[19,32]
Hypoxia-related pathways.	Hypoxia induces dormancy through the activation of HIF-1α and related signaling cascades.	HIF-1α, FBX8, NF-κB.	FBX8 regulates dormancy by modulating HIF-1α; promotes G0/G1 arrest and dormancy markers expression.	[5,12,19]
Stemness and dormancy overlap.	Dormant tumor cells share features and markers with cancer stem cells, suggesting overlapping mechanisms.	NANOG, SOX2, CD44, CD133, KLF4, AXIN2, LGR5, BMI1.	NANOG promotes dormancy via fatty acid oxidation and P21/P27 induction; other stemness factors mark dormant CSC-like.	[11,22,34,35,36,37]
Redox homeostasis.	Dormant cells maintain low ROS levels to evade oxidative damage and chemotherapy-induced apoptosis.	Antioxidant enzymes.	Reduces ROS below normal levels, avoiding ROS-mediated cell death and sustaining dormancy.	[30]
Dormant signature genes.	Shared gene expression patterns in dormant tumor cells across cancer types related to adhesion, TEM, TGF-b.	ZEB2, NANOG, factors related to cell adhesion and chemotaxis.	Regulate cell plasticity, migration, and dormancy across tumor types.	[10,11]
Cell cycle arrest regulation.	Induction and maintenance of cell cycle arrest at G0/G1 to maintain quiescence.	P21, P27, Cyclin D1, CDK4, c-MYC.	Cell cycle inhibitors P21 and P27 increase, Cyclin D1 and proliferative signals decrease, enforcing quiescence.	[5,11,32]

## 4. Potential Detection via Liquid Biopsy

### 4.1. Analysis of CTDNA and Circulating Tumor Cells (CTCs)

In CRC, ctDNA detection rates have been shown to correlate with disease stage. It is identified in 40% and more than 90% of patients with stage I and IV disease, respectively. Among patients with mCRC who underwent resection of liver metastases, the preoperative detection of peripheral blood-specific somatic genetic mutations was associated with poorer disease-specific survival [23]. One study evaluated peripheral blood samples from patients with mCRC after surgical resection. Cell morphology and kinetics between collection time points were considered in the survival analysis. We found that the CTCs, the CTC groups, and the apoptotic CTC groups indicate poor survival, with a progressive increase in cell count from before resection to some time after resection [24].

CTC detection and counting have been studied as prognostic markers in several types of cancer. One study evaluated the prognostic effect of CTCs detected by the CellSearch system (Menarini Group, Florence, Italy) in patients with CRC. An association between CTC detection and tumor stage was shown. Here, a higher CTC positivity rate was observed in patients with stage IV disease (60.7%) than in those with stage II–III disease (20.7–24.1%), and no CTCs were detected in the healthy population. The determination of CTC could represent a reliable substitute for tumor burden and the possibility of longitudinal disease profiling. A recent study determined whether the promoter of the gene SEPT9 methylated in a laboratory (analyzed in CTC) could be a potential biomarker for the early detection of CRC. Its detection through a real-time PCR assay was validated in a prospective study. However, the interpretation of the results depended largely on the relationship between the sensitivity and specificity of the study technique [28].

Thus, while CTCs and dormant cells are not equivalent, it may be possible to identify a specific molecular profile indicating that certain CTCs are in a dormant state. In fact, one study suggests that, although CTCs derived from colorectal cancer represent a heterogeneous population, a significant proportion exhibit features consistent with dormancy. This study used an orthotopic mouse model of mCRC with an HCT116 cell line to obtain CTCs. EpCAm-positive CTCs were isolated, and 19 genes related to epithelial phenotype, proliferation, stemness, migration, and immune evasion were analyzed by qPCR. A great decrease in the expression of epithelial and proliferation markers, such as ki67, was observed. On the contrary, an overexpression of two genes related to stemness, such as DLG7 and BMI1, was observed. In another study, CD47 overexpression was found to be related to immune system evasion. However, it was not possible to demonstrate the overexpression of this marker by the immunosuppression of the mice [36]. Therefore, the focus should be on the possibility of detecting these cells by studying these possible expression profiles.

In recent years, ctDNA has been extensively studied to identify MRD. A multicenter study evaluated the status of preoperative and postoperative ctDNA in stage I–III CRC. Thus, the detection of ctDNA made it possible to anticipate radiological relapse in approximately eight months. Based on this finding, ctDNA status was assessed in a cohort of 168 patients using a liquid biopsy of Signatory. The detection rate of ctDNA was significantly associated with the tumor stage and treatment response [38].

Following this approach to ctDNA analysis, another study has shown that 5-hydroxymethylcytosine (5 hmC) serves as a relatively stable DNA mark that performs distinct epigenetic functions. Active demethylation in the mammalian genome is mediated by the family of TET dioxygenases that transform 5-methylcytosine (5 mC) to 5-hydroxymethylcytosine (5 hmC) by oxidation until it is finally transformed into 5-carboxylysine (5 caC). Therefore, the “intermediate” 5 hmC is a mark of active demethylation [39,40].

As already discussed, TET2 is an epigenetic dioxygenase that controls dormancy status and is responsible for the enzymatic oxidation of 5 mC to 5 hmC. It was recently determined that 5 hmC could be a potential cfDNA biomarker for NSCLC and esophageal cancer. In addition, it was also shown that the 5 hmC of plasma cfDNA could distinguish CRC patients from healthy individuals with a sensitivity of 83% and a specificity of 94%. Thus, with this, a possible method of prognostic value in CRC could be established via liquid biopsy through the analysis of cfDNA and the determination of 5 hmC [21,32]. Therefore, cfDNA could also be considered as a possible biomarker of dormant cells or slow cycles [39,40].

### 4.2. Serum Analysis of Extracellular Vesicles (EVs)

Serum miRNAs encapsulated in EVs (EV-miRNA) have been studied as new non-invasive biomarkers for the diagnosis and prognosis of patients with mCRC treated with anti-VEGF. Baseline miRNA-21 and 92a were observed to exceed CEA levels in the diagnosis of 44 patients with mCRC compared to 17 healthy volunteers. In addition, patients who died had higher levels of miRNA-92a and 222 at 24 weeks after treatment. Therefore, high levels of miRNA-222 at 24 weeks were associated with lower overall survival. These data indicate that EV miRNAs have great potential as liquid biopsy biomarkers for the identification and prognosis of mCRC [3].

Following this line of research, it was also feasible to study the possibility of other miRNAs, such as miRNA-103 and miRNA-107, which have been seen to intervene in the regulation of some stem cells [41] since, in many respects, these cells share the same signaling mechanisms as quiescent cells. A possible approach could relate to whether some of these miRNAs also regulate dormant cells and can be detected in plasma associated with EVs.

This approach could focus on the NANOG factor, which, as we have already seen, is an important factor in cellular dormancy. According to a study, its regulation, among other factors, also depends on miRNAs [37]. Recent studies have provided compelling evidence that tumor cells can secrete exosomes containing specific microRNAs, such as miR-23b, which promote cellular dormancy and chemoresistance. For example, miR-23b has been shown to induce dormancy by downregulating a key regulator of cell proliferation and motility. This mechanism highlights the crucial role of exosome-mediated miRNA transfer in maintaining tumor cell quiescence and evasion from therapy [42].

This could open up more possibilities for searching for biomarkers, not only for prognosis but also for the detection and identification of these dormant cancer cells.

## 5. Possible Therapeutic Approaches

As for the study of treatment strategies against dormant cells, they have to be efficient enough to guarantee their complete elimination. The evidence accumulated to date suggests that persistent slow-cycling dormant cells may become more aggressive and confer a worse prognosis for the disease [12].

Possible strategies exist to eliminate these cells based on three mechanisms: awakening quiescent cells, maintaining quiescence, and eliminating quiescent cells.

### 5.1. Awakening Quiescent Cells

This mechanism involves “waking up” dormant cells, making them re-enter the cell division cycle. This allows for their elimination using the antiproliferative drugs. Identifying the signaling pathways needed to maintain a low proliferation rate or quiescent state could facilitate the design of effective “wakening” treatments that could be combined with antiproliferative therapies to prevent cancer relapse [12].

Since cancer stem cells (CSCs) and quiescent tumor cells share signaling pathways, as in the case of mitogen-activated protein kinase P38 (P38 MAPK), the development of therapeutic approaches targeting p38 may increase tumor sensitivity to chemotherapy and prevent progression and metastasis. Thus, the inhibition of p38 by SB203580 in combination with 5-Fluoruracil (5-FU) significantly reduces cell viability and increases cell death and caspase activity by increasing BAX expression. It also improves the sensitivity of CRC cells to irinotecan, which alone results in P38 activation by raising phosphorylated P38 levels [32,33].

While it appears that reactivating quiescent cells as part of a “wakening” strategy could overcome resistance to chemotherapy drugs, the clinical implementation of this strategy is likely to pose a challenge. It is difficult to ensure that all cells re-enter the cell cycle so that they can be eliminated. Recent studies suggest that such strategies could rapidly drive tumor recurrence and worsen patient outcomes in some cases [32,33].

### 5.2. Maintenance of Quiescence

Another treatment possibility is based on maintaining quiescence, thus avoiding proliferation and rapid tumor growth. Since moving from a resting state to a proliferative state led to worse outcomes, it seems that keeping them in the quiescent state may be a better approach to prevent tumor recurrence. As mentioned above, dormant cells have an imbalance in the ratio between P38 and ERK (Figure 2). Strategies to modulate the P38/ERK pathways could lead to a permanent halt of quiescent cell growth, preventing tumor recurrence and metastasis [12]. Therefore, if we can precisely modulate the balance of P38 and ERK, we will be able to induce permanent inactivity and prevent metastasis, which will mark a new era in cancer treatment [20].

Thus, this work studied how dormant tumor cells “wake up” once they reach the target organ. They found a new combination of growth factors and cytokines produced by ECM cells. The binding of fibronectin to integrins has a critical role in changing the balance of P38 and ERK activity in favor of ERK. In addition, chronic inflammation can induce the growth of these cells again through the activation of integrins. Macrophages promote fibronectin secretion by fibroblasts by favoring fibronectin binding to the integrins of dormant cells. Therefore, molecules involved in the “wakening” of dormant tumor cells are being studied to develop strategies to keep them in a dormant state for cancer treatment, either as single or combined agents [21].

Following this line of dormancy maintenance, another study focused on the possibility of using NR2F1 agonists (member 1 of group F of the nuclear receptor subfamily 2, also known as COUP-TF1) to induce quiescence as a therapeutic strategy to prevent metastasis. In this study, epigenetic reprogramming therapy using a DNMT1 inhibitor, 5-azacitidine (AZA), and retinoic acid (RA) resulted in the overexpression of NR2F1 and induction of quiescence in several cancer models. Therefore, NR2F1 activation was tested with a small molecule agonist to induce and maintain dormancy, preventing recurrence and metastasis. In addition, the antimetastatic effect could persist even after treatment discontinuation, indicating a durable reprogramming of the malignant cells in the quiescent state [43].

Another possibility that could be considered as a treatment target at this point is FBX8, which, as already mentioned in the characterization of dormant cells, intervenes in the degradation of HIF-1α, CDK4, and c-MYC, inhibiting angiogenesis and cell cycle and proliferation. Thus, using drugs capable of maintaining high levels of FBX8 for a prolonged period, either by promoting overexpression or by inhibiting its degradation, it would be possible to maintain cell dormancy and control metastasis.

However, all of these possible strategies require maintaining the quiescent state for a long time to prevent the tumor from regrowing, which can be very difficult to achieve given the high adaptability of cancer cells to different scenarios. In addition, to keep the cells dormant for a long period, permanent treatment is needed, which seems clinically unfeasible mainly due to toxicity. In addition, long-term treatments can always generate resistance, further complicating the approach to tumor recurrence [12].

### 5.3. Eliminate Quiescent Cells

The third therapeutic approach against these quiescent cells involves specific treatments to directly eliminate the cells in a quiescent state. For instance, one study proposes that quiescent cells in CRC could also be eliminated using autophagy. Dormant cells treated with inhibitors of ULK1, a crucial kinase that activates autophagy, in combination with standard chemotherapy treatment, did not regrow and underwent apoptosis, even after treatment discontinuation [13].

Another possibility focuses on inhibiting the transcription factor NANOG that plays important roles in the development of drug resistance, migration, and the state of tumor cell dormancy. Khosravi N et al. evaluated the suppression effect of NANOG using small interfering RNA (siRNA) combined with 5-fluorouracil (5-FU) in CRC cells [34]. SW-480 cells overexpressing NANOG were transfected with siRNA and treated with 5-FU, in combination or separately. Subsequently, it was observed that NANOG expression was significantly reduced after transfection of SW-480 cells using NANOG siRNA at mRNA and protein levels. In addition, NANOG clearance significantly increased the sensitivity of CRC cells to the drug 5-FU through modulation of Bax and Bcl-2 mRNA expression. NANOG removal and 5-FU treatment decreased the ability of cells to migrate and self-renew. The combination therapy led to cell cycle arrest in the sub-G1 phase in CRC cells. Taken together, the results indicated that NANOG plays an important role in the drug resistance, migration, and self-renewal of CRC cells. Therefore, reducing NANOG levels would also be a promising target in combination with 5-FU for the development of new therapeutic approaches to CRC [34].

A recent study evaluated the effect of Orlistat (a drug normally used to treat obesity) on the gene expression of OCT4, NANOG, SOX2, and KLF4 in the CRC cancer cell line SW40. Expression changes at the mRNA level of OCT4, NANOG, KLF4, and SOX2 were determined by real-time PCR. The mRNA expression of all these factors was significantly reduced after the treatment of SW40 cell lines with 100 μM doses of Orlistat. Therefore, Orlistat could also be used to treat CRC [35].

The unique biomarkers identified in dormant cells could be a potential target for the immune system. Chimeric antigen receptor (CAR) T cells could be a potential candidate to directly recognize and eliminate quiescent tumor cells. Therefore, there is an urgent need to discover surface markers of dormant cells and their detection by specific labeling in vivo [12]. Some of the surface markers shared by dormant cells and cancer stem cells, CD133 and CD44, could be potential targets of CAR-T cells. CAR-T cells can eliminate dormant cells by directly targeting these surface markers.

Strategies targeting microRNAs (miRs), either by inhibiting oncogenic miRs or replacing tumor-suppressor miRs, are promising in cancer therapy. Due to their nanocarrier properties, exosomes are being explored as delivery vehicles for anti-miRs, chemotherapy, or ATP to overcome chemoresistance. For instance, MRX34, a liposome-encapsulated miR-34 mimic, downregulates the oncogenic miR-21, inhibiting tumor growth and metastasis. These approaches could also be applied as therapies against dormant cancer cells, aiming to disrupt their quiescence and resistance mechanisms [42].

## 6. Conclusions

While the survival of CRC has increased in recent years, it is still uncommon since its risk of recurrence and metastasis remains high. Specifically, for patients with CRC and liver metastases who achieve potentially curative surgeries, the early detection of recurrence is crucial. The main challenge is that a significant percentage of these patients suffer from relapses. Currently, the detection method based on imaging techniques and markers, such as CEA and CA19-9, is inefficient regarding early detection.

Therefore, it is necessary to identify and characterize the cells involved in these relapses that escape chemotherapy treatments. Identifying them using minimally invasive diagnostic methods with sufficient sensitivity and specificity is important in routine clinical practice.

After reviewing the current literature, few studies have determined the signaling pathways involved in inducing cell dormancy. All these pathways allow these cells to express stem markers and factors to reduce proliferation.

Diagnosis has been improved with new methods based on liquid biopsy, mainly on the analysis of cfDNA and CTC for the detection of markers compared to the usual biochemical markers CEA and CA19-9. While there are still studies with contradictory results regarding the value of using these markers in liquid biopsy to detect recurrences in the follow-up of patients, most of these studies have similar results compared to tissue biopsy. Therefore, they have great potential for their application in the follow-up of patients. While there are no established biomarkers to specifically detect dormant cells using liquid biopsy, the mechanisms underlying the induction of the dormant state and the factors that seem to be key in maintaining this state are increasingly known. This opens up a great field of exploration so that these potential biomarkers can be established in clinical practice for the early detection of these cells and to prevent the progression of the disease.

Since dormant cells seem to share common signaling pathways with cancer stem cells, possible biomarkers of these stem cells could be studied, and some method to differentiate them from dormant cells could be sought. This would make it easy to predict the risk of metastasis and recurrence in patients with mCRC. As aforementioned, DLG7 and BMI1 have been detected in positive EpCAM CTCs in mCRC cell lines in mouse models.

Treatment against cell dormancy also seems quite promising. However, it depends on the correct characterization of dormant cells with clear biomarkers that allow for their possible use as therapeutic targets. Possible strategies exist to eliminate dormant cells based on three mechanisms: awakening quiescent cells, maintaining quiescence, and eliminating quiescent cells. The most promising strategy is the direct elimination of these quiescent cells through the modulation of the main factors and mechanisms involved in the regulation of this state of dormancy. A good case in point is the use of Orlistat and CAR-T cells, which seem to be a promising treatment option for patients with mCRC.

The main limitation of this literature review is attributed to the difficulty in finding universal biomarkers for their application in routine clinical practice.

## Data Availability

Not applicable.
